# Identification and practical validation of spectrally efficient non-orthogonal frequency shaping waveform

**DOI:** 10.1038/s44172-023-00108-w

**Published:** 2023-08-19

**Authors:** Tongyang Xu, Izzat Darwazeh

**Affiliations:** 1 School of Engineering, Newcastle University, Newcastle upon Tyne, NE1 7RU UK; 2 Department of Electronic and Electrical Engineering, University College London, London, WC1E 7JE UK

**Keywords:** Electrical and electronic engineering, Information technology

## Abstract

Signal waveform is the basic physical layer element that fundamentally determines the most important key performance indicator in communication systems, namely spectral efficiency. Traditional waveforms rely on orthogonal Nyquist shaping but result in restricted spectral efficiency. Non-orthogonal waveforms have been proposed to enhance spectral efficiency via compressing either time or frequency resources. However, beyond 25% efficiency improvements in this way, classical Mazo theory states that performance will start to degrade. Here we use a robust and low-complexity neural network modulator to create asymmetric sub-carrier shapes, for multi-carrier non-orthogonal frequency shaping (NOFS) signals. The result is a non-Nyquist waveform which achieves 150% spectral efficiency improvement and still operates well with simple receiver processing. We set up a hardware communication link with over-the-air image transmission that practically validates the spectral efficiency improvement. This spectral efficiency will save communication resources for future communications systems such as 6G services.

## Abstract (web summary)

Tongyang Xu and Izzat Darwazeh use machine learning to identify a non-Nyquist waveform with high spectral efficiency, which is then practically validated with over-the-air image transmission using a hardware communication link. Waveforms such as these and the resulting spectral resource saving could be useful for enabling future 6G communications systems.

## Introduction

The quest of maximizing the rate of information transfer over limited physical channels has been with us for nearly 100 years. Harry Nyquist, working in the early 1920s on optimizing the performance of telegraph systems, published a paper in 1924^1^, which defines optimum signal shapes to be used over band limiting telegraph wires. Nyquist inter symbol interference (ISI) criteria, as well as his 1928 published sampling theorem^2^, are the foundations in all of today’s communication systems^3–7^.

Nyquist signals are termed ‘orthogonal’ in that they do not interfere with each other at the time instant that matters and that is the point at which the bit is sampled to decide whether it is a binary ‘1’ or a binary ‘0’. The concept was transformed in 1957 by^8^ to the frequency domain with signals that are orthogonal in frequency to allow transmission of high bit rate data over cables connecting different parts of computing systems. In 1966, yet again at Bell Labs, Chang^9^ patented a similar concept and termed it orthogonal frequency division multiplexing (OFDM). The method used Nyquist shaped frequency domain signals, each modulating one of a set of sinusoidal carriers termed sub-carriers, resulting in zero interference, and hence orthogonal. OFDM is now the method of choice of many systems and has been standardized in 4G, 5G, wireless local area network (WLAN)^10^, Worldwide interoperability for Microwave Access (WiMAX)^11^ and digital video broadcast (DVB)^12^.

50 years after Nyquist, also in Bell Labs in 1975, J. E. Mazo, aimed to discover through mathematical modelling the limits of Nyquist criterion. Mazo argued that by trading off bit rates versus system performance, described in error rates, Nyquist criterion may be relaxed and higher bit rates can be transmitted over a band limited channel. Detailed studies reported in Mazo’s classic 1975 paper^13^ showed that Nyquist criterion may be relaxed; bit rates may be increased by a factor of 25% without compromising the received signal quality, provided Nyquist pulse shapes are used and an optimum detector is used at the receiver. Since then, several groups worldwide worked on non-orthogonal systems such as spectrally efficient frequency division multiplexing (SEFDM)^14–16^, faster than Nyquist (FTN)^17,18^, Fast-OFDM^19^, generalized frequency division multiplexing (GFDM)^20^ and filter bank multicarrier (FBMC)^21^.

With the advancement of 5G and future 6G^22–26^, artificial intelligence (AI) is finding applications in signal communications both in wireless^27–29^ and wired^30,31^ domains. For transmitter side AI applications, machine learning is commonly used for multiple input multiple output (MIMO) precoding^32,33^, beamforming and beam-steering^34^. For the receiver side, more applications are possible such as modulation classification^35–37^, signal detection^38,39^ and channel estimation^40^. For machine learning applications aiming for both transmitter and receiver sides, end-to-end autoencoder^41,42^ is the representative technique. Therefore, AI is a potential tool to assist communications especially for the design of new signal waveforms.

In this article, we address the limitations of all current systems and report an advanced waveform design termed nonorthogonal frequency spacing (NOFS). We use machine learning techniques to create ‘Asymmetric’ structured sub-carriers, shaped by irregular-Sinc (irSinc), for multi-carrier NOFS signals, different from Sinc and non-Sinc Nyquist signals, in that NOFS can improve spectral efficiency by 150%. Our approach designs a robust and low-complexity neural network modulator to create new shapes for each sub-carrier. Hence, instead of using Sinc shaped sub-carrier for Nyquist signals, the proposed NOFS is a non-Nyquist signal format with irSinc shaped sub-carriers. Each irSinc shaped sub-carrier can be independently tuned to mitigate signal waveform internal interference using machine learning techniques, with the trade for wider out-of-band power leakage. To validate our theoretical discoveries, we build a communication hardware testbed with antennas integrated for over-the-air signal transmission. Importantly, we achieved the world-first 150% spectral efficiency improvement record via over-the-air image transmission, which indicates the potentials of the waveform in practice and opens the era of multi-fold spectral efficiency increase using the newly proposed non-Nyquist waveform design.

## Results and Discussions

### Non-orthogonal waveform basics

For the commonly used OFDM waveform, time and frequency orthogonality are the key signal features. A general OFDM signal, modulated by *N* sub-carriers, is expressed in the following

1 $${x}_{k}=\frac{1}{\sqrt{N}}\mathop{\sum }\limits_{n=1}^{N}{s}_{n}\exp \left(\frac{j2\pi nk}{N}\right),$$

where *x*_*k*_ is the *k* ^*th*^ time-domain sample with the index of *k* = 1, 2, . . . , *N* and *s*_*n*_ is a single-carrier symbol modulated on the *n* ^*th*^ sub-carrier in an OFDM symbol.

In 1975, J.E.Mazo^13^ proved that data can be sent faster than what is set by Nyquist rate, effectively taking only 80% of time resources, without performance loss when optimum processing of the received waveforms is assumed. The spectral efficiency is therefore improved by 25% computed as (1/0.8 − 1) × 100. The Mazo theory is applicable in both time-domain FTN and frequency-domain SEFDM waveforms because improving spectral efficiency, defined by *S*_*E*_ = *R*_*d*_/*B*, could be achieved by either sending data faster (i.e. increase *R*_*d*_) or compressing occupied spectral bandwidth (i.e., reduce *B*). Due to the spectrum scarcity, here we focus on the spectral resource saving waveform SEFDM, by generalizing the expression in (1) to become:

2 $${x}_{k}=\frac{1}{\sqrt{N}}\mathop{\sum }\limits_{n=1}^{N}{s}_{n}\exp \left(\frac{j2\pi nk\alpha }{N}\right),$$

where *α* = Δ*f* ⋅ *T* is the sub-carrier spacing compression factor, Δ*f* is the sub-carrier spacing and *T* is the time duration of one SEFDM symbol. It is apparent that the SEFDM signal expression in (2) is similar to OFDM but with an additional variable 0 < *α* < 1.

**Fig. 1:**
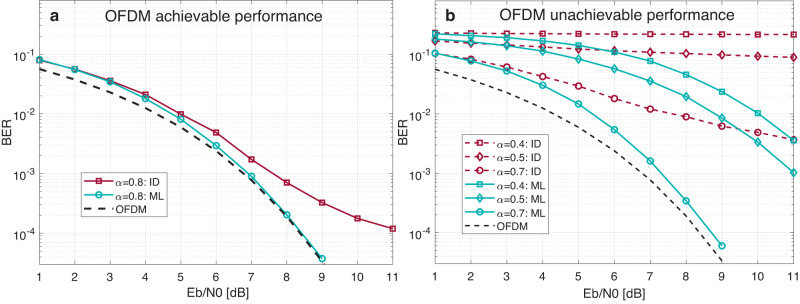
Uncoded performance of conventional non-orthogonal signals. Spectral bandwidth compression factor is *α*; Spectral bandwidth saving is *P*_*BS*_ = (1 − *α*) × 100%; Spectral efficiency improvement is *P*_*SE*_ = (1/*α* − 1) × 100%. Optimal signal detection: maximum likelihood (ML); Sub-optimal signal detection: iterative detection (ID). Modulation format: quadrature phase shift keying (QPSK). **a** Orthogonal frequency division multiplexing (OFDM) achievable performance when the bandwidth compression factor is *α* = 0.8 (spectral resource is saved by *P*_*BS*_ = 20% and spectral efficiency is improved by *P*_*SE*_ = 25%). **b** OFDM unachievable performance where three factors are evaluated. *α* = 0.7 (spectral resource is saved by *P*_*BS*_ = 30% and spectral efficiency is improved by *P*_*SE*_ = 43%). *α* = 0.5 (spectral resource is saved by *P*_*BS'*_ = 50% and spectral efficiency is improved by *P*_*SE*_ = 100%). *α* = 0.4 (spectral resource is saved by *P*_*BS*_ = 60% and spectral efficiency is improved by *P*_*SE*_ = 150%).

Figure 1 demonstrates the bit error rate (BER) performance for frequency-domain SEFDM waveforms when OFDM performance is considered as the comparison benchmark. Details of the signal detection algorithms in Fig. 1 are provided in Methods. Figure 1a evaluates OFDM achievable performance when the non-orthogonal signal is compressed with *α* = 0.8. Results show that the maximum likelihood (ML) detection, which is the optimal solution defined in (10), can push the performance close to the ideal OFDM performance. The sub-optimal iterative detection (ID) method^43^ shows divergent performance but with greatly reduced signal processing complexity. For compression ratios below *α* = 0.8 in Fig. 1b, the optimal ML detection fails when three spectral compression factors are evaluated. Firstly, we test *α* = 0.7 because this factor is smaller than *α* = 0.8. Results show that the SEFDM signal cannot be completely recovered even with the optimal ML signal detection. Then, we test *α* = 0.5, which is the Fast-OFDM compression ratio^19^ where only one-dimensional modulation formats can work at such a high compression ratio. Results are also expected that the ML detector fails to recover two-dimensional QPSK signals. We further compress the ratio to *α* = 0.4, which is the factor beyond Fast-OFDM compression ratio with expected results of failing to recover signals. The performance loss comes from increased inter carrier interference (ICI), associated with the loss of orthogonality, when spectral resources are densely compressed. Here, even the optimal ML signal detector can not recover signals. This work therefore aims to explore the challenge and propose new solutions relying on an integrated waveform and intelligence framework to improve spectral efficiency go beyond the *α* = 0.8 limit.

### Non-orthogonal frequency shaping waveform

The sub-carrier component $\exp \left(\frac{j2\pi nk\alpha }{N}\right)$ in (2) contains unique information in time and frequency. It is expected that by tuning each time-frequency component, new signal features would be obtained. However, there is no guiding principle on how to modify each time-frequency component since randomly modifying each element may cause unexpected time-frequency information violation.

Neural network can learn and tune internal neuron connections via continuous training. Therefore we propose in this work to use neural networks to emulate sub-carrier characteristics and then optimize each time-frequency component via continuous training. A general multi-layer neural network architecture is mathematically expressed as

3 $${a}_{i}^{(m+1)}=\sigma \left(\mathop{\sum}\limits_{j}{w}_{ij}^{(m+1)}{b}_{j}^{(m)}+{\psi }_{i}^{(m+1)}\right),$$

where ${a}_{i}^{(m+1)}$ is the *i* ^*th*^ output element at the (*m*+1)^*th*^ layer, ${b}_{j}^{(m)}$ is the *j* ^*th*^ input element at the *m* ^*th*^ layer, ${w}_{ij}^{(m+1)}$ represents a weight element at the (*m*+1)^*th*^ layer, ${\psi }_{i}^{(m+1)}$ indicates the *i* ^*th*^ bias element at the (*m*+1)^*th*^ layer and *σ*( ⋅ ) is an activation function. By tuning the number of network layers and the number of neural elements at each layer, a neural network can realize complex functions.

To make the designed neural network functionally similar to the multicarrier signal expressions in (1),(2), the general multi-layer neural network architecture in (3) is simplified into a one-layer neural network as the following

4 $${x}_{i}^{(2)}=\sigma \left(\, \mathop{\sum }\limits_{j=1}^{N}{w}_{ij}^{(2)}{s}_{j}^{(1)}+{\psi }_{i}^{2}\right),$$

where the input vector *s* ^(1)^ and output vector *x* ^(2)^ of the neural network are both scaled to have *N* elements, representing the input and output relationship in (1),(2). To further simplify the expression in (4), the neural network based signal generation is obtained in a matrix format as the following

5 $${{{{{{{\bf{x}}}}}}}}=\sigma ({{{{{{{\bf{W}}}}}}}}{{{{{{{\bf{s}}}}}}}}+\Psi ),$$

where the role of the matrix **W** is similar to the sub-carrier modulation in (1),(2). Therefore, modifying elements in **W** is equivalent to changing time-frequency information of the sub-carrier modulator leading to new sub-carrier features. The elements in **W** represent the weights of neural connections and their internal connections indicate sub-carrier characteristics and sub-carrier spacing compression factors. A large value of weight in **W** indicates that the neural connection plays an important role. On the other hand, a small weight indicates a less important connection, which could be discarded to mitigate ICI and cut signal processing complexity. It is believed that optimizing the internal neural connections can let the matrix **W** make the most use of neural network learning capability and leading to the optimal signal generation.

We introduce a pruning factor *γ* to determine important neural connections and less important neural connections. For those less important neural connections, we can easily discard them since their disappearance will have no great effect to the network performance. After re-training the pruned neural network, a sparsely connected NOFS neural modulation matrix **W**_**γ**_ is obtained with optimized time-frequency components. Hence, the NOFS signal, generated by the neural modulator, is expressed as

6 $${\bf{x}}_{NOFS}=\sigma ({\bf{W}}_{{\boldsymbol{\gamma }}}{\bf{s}}+\Psi ).$$

Therefore, by continuously training the neural network with proper configurations in **W**_**γ**_, Ψ and *σ*( ⋅ ), an NOFS signal with bespoke features is obtained. The irSinc shape is highly adaptive and will change when the signal configuration changes. Therefore, it is not described here mathematically nor is derived following information theoretic approaches. This is in contrast to classical signal formats such as OFDM, SEFDM or FTN, which are commonly described using deterministic mathematical expressions such as using the classical Fourier transform. Thus, the viable solution to define NOFS signals is still the learning based neural network expression in (6). The size of the matrix **W**_**γ**_ indicates the number of sub-carriers and the elements within **W**_**γ**_ and all other changeable parameters jointly determine the effective compression factor.

### irSinc shaping

**Fig. 2:**
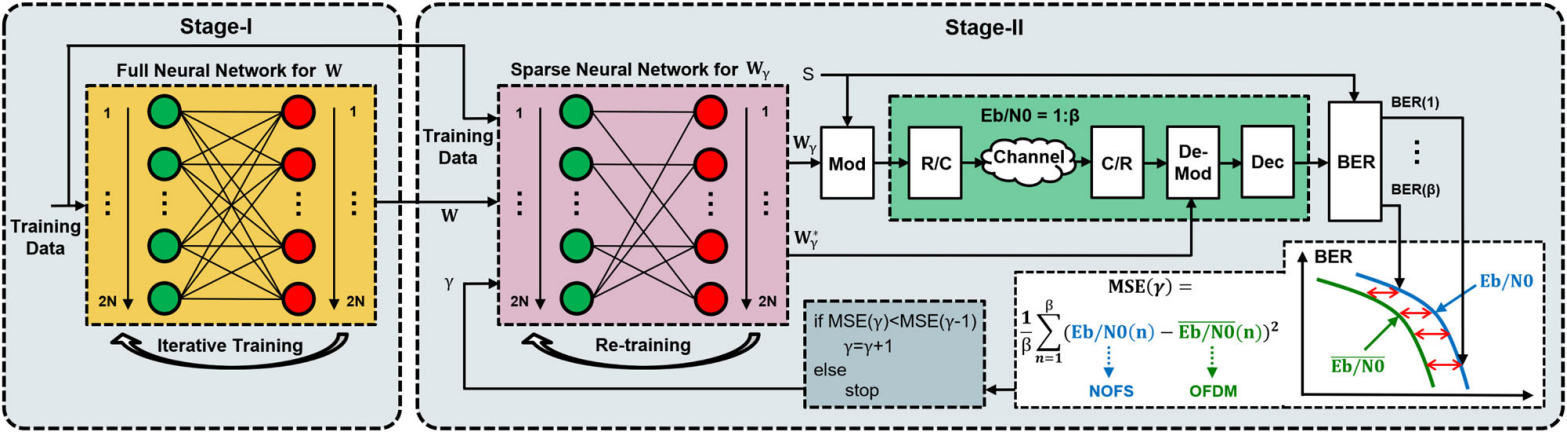
NOFS signal waveform training framework. Mod Modulation, R/C Real to complex conversion, C/R Complex to real conversion, De-Mod Demodulation, Dec Detection, BER Bit error rate, MSE Mean squared error, OFDM Orthogonal frequency division multiplexing, NOFS Non-orthogonal frequency shaping.

The NOFS training framework is illustrated in Fig. 2, with a complete transmission and reception link. The training process is separated into two stages; Stage-I concerns pre-processing and Stage-II focuses on the NOFS neural modulator training. Details of the training methodology is provided in Methods, and the final aim of the training is to obtain the neural modulator matrix **W**_**γ**_ that can enable the best performance for NOFS signals.

**Fig. 3:**
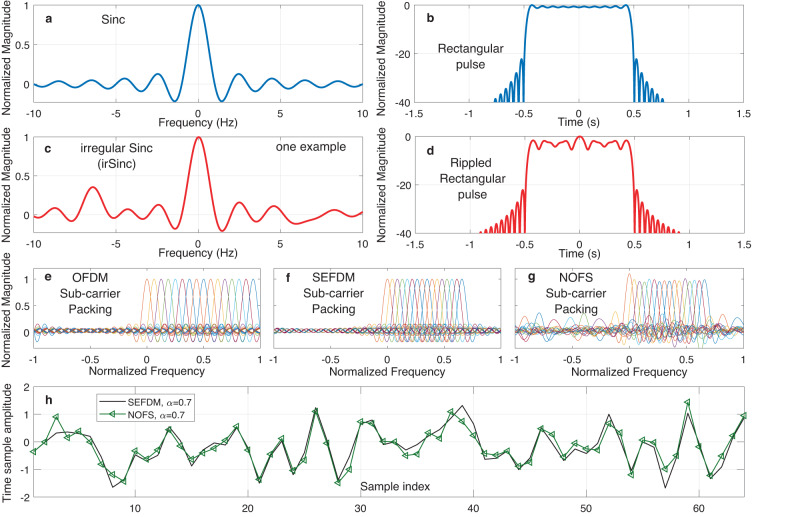
Comparisons of traditional Sinc frequency response and irSinc frequency response with their time-domain pulses. **a** Traditional Sinc frequency response. **b** Time-domain rectangular pulse corresponding to the traditional Sinc frequency response. **c** irSinc frequency response (one example). **d** Time-domain rippled rectangular pulse corresponding to the irSinc frequency response. **e** Sub-carrier packing pattern for orthogonal frequency division multiplexing (OFDM). **f** Sub-carrier packing pattern for spectrally efficient frequency division multiplexing (SEFDM). **g** Sub-carrier packing pattern for non-orthogonal frequency shaping (NOFS). **h** Time-domain samples comparison between SEFDM and NOFS signals.

In conventional communication systems, a rectangular pulse is commonly used in time-domain, such that its frequency response has a Sinc function shape. An ideal Sinc is unachievable in practical systems due to its doubly infinite frequency response, therefore a truncated Sinc is applied in practice such as in 4G, 5G and WiFi standards. We show a truncated version of a Sinc frequency response in Fig. 3a. To simplify the illustration, we normalize the Sinc frequency response and place it at 0 Hz center frequency with frequency crossing at [ − 10, − 9, . . . , − 2, − 1, 1, 2, . . . , 9, 10] Hz. Correspondingly, its time-domain pulse is in a rectangular shape in Fig. 3b with a 1 s rectangular pulse duration defined between -0.5 s and 0.5 s. It is noted that by changing the roll-of-factor of a rectangular pulse (from zero), its Sinc spectrum changes to a non-Sinc, such as that of raised cosine pulse spectrum, commonly used with single-carrier signals but not always used in non-orthogonal multicarrier signals, where limited performance enhancement will be expected^44^, given the already existent intercarrier interference. Many non-Sinc or non-T-orthogonal pulses, where there is no requirement for zero spectral crossing at the inverse of the symbol period, as elegantly detailed in Chapter 7 of^45^, are designed mainly for single-carrier signals. The key objective is to cut out-of-band power emission, in other words limit the signal band, as described by Slepian in his classic 1976 note “On Bandwidth”^46^, while minimally compromising their time domain inter symbol interference. Work in^47^ is based on applying numerical methods to derive frequency domain pulse spectra/filter responses, optimised for specific modulation formats so as to result in non-T-orthogonal pulses that minimize bandwidth. Instead of optimizing out-of-band power emission for single carrier systems in other available works, our design here aims for multicarrier systems such that each subcarrier is designed with a unique irSinc shape leading to the mitigation of signal intercarrier interference.

As explained in the previous section, the tuning of the neural modulation matrix **W**_**γ**_ enables new features for sub-carriers. A unique sub-carrier design termed irregular Sinc (irSinc) is demonstrated in Fig. 3c where an asymmetric frequency response is illustrated as one example. The frequency crossing is no longer strictly at [ − 10, − 9, . . . , − 2, − 1, 1, 2, . . . , 9, 10] Hz, which indicates irregular frequency response characteristics compared to the symmetric Sinc. In terms of the time-domain pulse in Fig. 3d, unlike the flat rectangular pulse in Fig. 3b, the newly designed irSinc results in a rectangular pulse with ripples at the top. Regardless of these ripples, the rippled rectangular pulse in Fig. 3d is still defined between −0.5 s and 0.5 s. Since sub-carriers are shaped by irSinc rather than the traditional Sinc, it is expected to achieve the goal of higher spectral efficiency improvement more than 25% via the use of the irSinc design.

The spectra for traditional OFDM and SEFDM in Fig. 3e, f utilize evenly packed Sinc shaped sub-carriers with the same features per sub-carrier in terms of sub-carrier bandwidth, shape and magnitude. However, for our proposed NOFS waveform, sub-carriers are not uniform and each sub-carrier is shaped by an independent irSinc with variations in sub-carrier bandwidth, shape and magnitude, leading to non-orthogonal frequency shaping in Fig. 3g. The advantage of using such a waveform is to have more design flexibility since each sub-carrier is shaped by independent irSinc and is no longer constrained by the traditional Sinc shaping. One potential trade-off from the design is its increased out-of-band power leakage due to the irregular shape of irSinc. Therefore, the re-training operation at Stage-II in Fig. 2 aims to balance the spectral resource saving performance and increased out-of-band power leakage by re-training the sparse neural network using original training data.

The neural modulator training in Fig. 2 requires signal approximation, neural connection pruning and re-training. To obtain new signal features, our proposed modulator training framework aims to minimize the gap between NOFS BER and OFDM BER rather than minimizing the difference between inference signals and training signals. Therefore, advanced signal features could be obtained but at the cost of losing original signal characteristics. In Fig. 3h, the time-domain samples from NOFS are compared with the original samples from SEFDM showing sample sequence difference.

### Verification and characterization of NOFS

To show the advantages of NOFS in maintaining performance and saving spectral resources, we select OFDM and SEFDM as the benchmarks and list the numerical achievements of NOFS in Table 1. The choice of *α* is flexible and this work considers *α* = 0.7, 0.5, 0.4, with corresponding spectral bandwidth saving (*P*_*BS*_ = (1 − *α*) × 100%) of 30%, 50% and 60%. Accordingly, the spectral efficiency improvement (*P*_*SE*_ = (1/*α* − 1) × 100%) can reach 43%, 100% and 150%.

**Table 1:** Spectral efficiency improvement of NOFS compared to OFDM.

Waveform	Compression *α*	*η* (bit/s/Hz, QPSK)	Improvement
OFDM	1	2	0
NOFS	0.7	2.86	43%
NOFS	0.5	4	100%
NOFS	0.4	5	150%

**Fig. 4:**
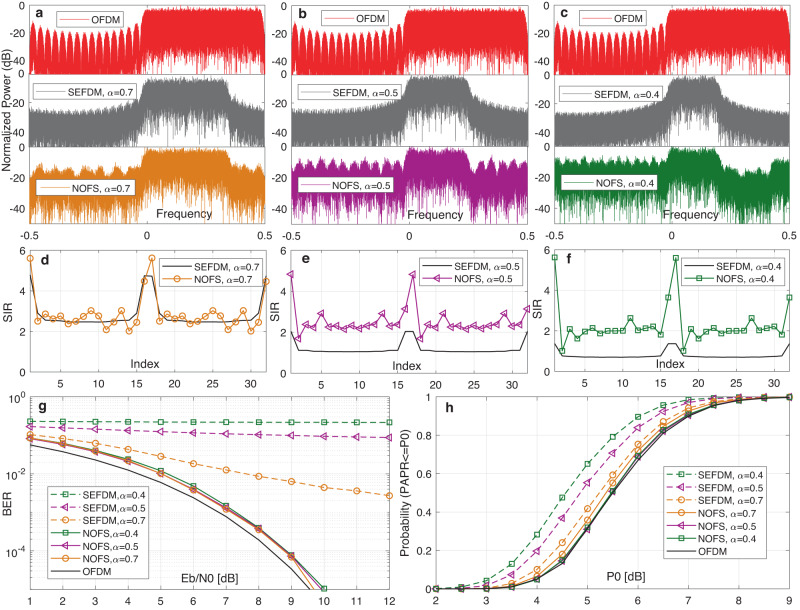
Comprehensive performance evaluations of NOFS signal waveform with *P*_*BS*_ = 30%, *P*_*BS*_ = 50% and *P*_*BS*_ = 60% spectral bandwidth saving. **a** Spectral response for signal waveforms with *P*_*BS*_ = 30% bandwidth saving. **b** Spectral response for signal waveforms with *P*_*BS*_ = 50% bandwidth saving. **c** Spectral response for signal waveforms with *P*_*BS*_ = 60% bandwidth saving. **d** Signal-to-interference ratio (SIR) analysis for inter carrier interference (ICI) mitigation impact on signal waveforms with *P*_*BS*_ = 30% bandwidth saving. **e** SIR analysis for ICI mitigation impact on signal waveforms with *P*_*BS*_ = 50% bandwidth saving. **f** SIR analysis for ICI mitigation impact on signal waveforms with *P*_*BS*_ = 60% bandwidth saving. **g** Bit error rate (BER) performance for each signal waveform. **h** Peak-to-average power ratio (PAPR) performance for each signal waveform. OFDM Orthogonal frequency division multiplexing, SEFDM Spectrally efficient frequency division multiplexing, NOFS Non-orthogonal frequency shaping.

The spectral resource occupation of NOFS is similar to SEFDM but is greatly reduced relative to OFDM as compared in Fig. 4a–c where the spectral peak power is normalized to 0dB. However, one critical impact from the NOFS waveform is its increased out-of-band power leakage due to the use of irSinc. The power of the out-of-band part will be increased with the increased level of ICI. The high out-of-band power leakage limits the signal application scenarios but it should not be considered as the signal bandwidth occupancy. Unlike pulse shaping signals, such a high out-of-band power leakage signal design is not suitable for omni-directional co-existence communication scenarios because it would cause interference to adjacent signals. For future communications, directional transmission link will be the dominated technique where antenna array or directional antenna can generate narrow beams^48^ to specific users and therefore inter-user interference can be avoided. In such cases, the out-of-band power leakage from NOFS is not a limiting factor anymore and should not be counted as the occupied bandwidth. In addition, for short and medium distance communications, signals with short wavelength at high carrier frequencies^49^ such as mmWave and THz are commonly used together with beamforming where a directional beam^50^ is shaped to point at the receiver. In this case, signal power is focused to the target and power leakage is prevented such that the out-of-band power leakage from NOFS would not interfere with other user signals. For long distance transmission scenarios, received signal power is weak and thus the noise power may be higher than its out-of-band leakage power. Given the above explanations, the out-of-band power leakage is not a limiting factor when proper application scenarios are considered. Therefore, it is believed that the effective signal bandwidth still depends on the in-band signal.

It is noted that ICI is caused by non-orthogonally packed sub-carriers, which could be indicated by non-diagonal elements within a correlation matrix between sub-carriers and their conjugate. The correlation between the neural modulation matrix **W** and its conjugate **W** ^*^ in NOFS is defined as

7 $${{{{{{{\bf{{C}}}}}}}_{\gamma }}}={{{{{{{\bf{{W}}}}}}}_{\gamma }^{* }}}{{{{{{{{\bf{W}}}}}}}}}_{{{{{{{{\boldsymbol{\gamma }}}}}}}}}.$$

Intentional deleting of neural connections and their associated weights could create advanced features in **W**_**γ**_ and therefore mitigate ICI. To have a general idea of ICI characteristics in NOFS, we use signal-to-interference ratio (SIR) below to demonstrate the ICI variations.

8 $$SIR(k)=\frac{{[{c}_{\gamma }(k,k)]}^{2}}{\mathop{\sum }\nolimits_{n = 1,n\ne k}^{2N}{[{c}_{\gamma }(k,n)]}^{2}},$$

where *S* *I* *R*(*k*), 1 < *k* < 2*N* indicates the *k* ^*th*^ SIR considering interference from all other sub-carriers. It is apparent in Fig. 4d–f that the SIR of SEFDM decreases with the reduction of *α*. However, for NOFS waveforms, the SIR almost maintains at the same level even when *α* is reduced, indicating the effectiveness of mitigating ICI by tuning the neural modulation matrix **W**_**γ**_.

For practical implementation and power efficiency considerations, we mainly use the low-complexity ID detector (see Methods for details) to test the performance of our new waveforms, instead of the high-complexity ML detector (see Methods for details). In Fig. 4g, all the SEFDM signals show great BER degradations compared to the OFDM performance while our proposed NOFS waveforms show approaching performance to OFDM, even when signals are compressed more than *α* = 0.8.

In Fig. 4h, it is obvious that OFDM has the worst peak-to-average power ratio (PAPR) performance while SEFDM shows the best PAPR performance, which indicates the advantage of signal non-orthogonality in achieving better PAPR. For NOFS waveforms, the irSinc shaped sub-carriers obviously affect PAPR but in a ‘good’ way where all the signals show better PAPR than OFDM. With the increase of *α*, the PAPR becomes better and approaches SEFDM. When *α* is reduced, the PAPR becomes worse but still outperforms OFDM. It is noted that the objective of training or optimization of irSinc in NOFS is to cut intercarrier interference rather than improving PAPR or cutting out-of-band power. Therefore, the better PAPR achieved by using irSinc is an additional benefit and this work does not intentionally optimize PAPR.

### Over-the-Air validation and application in image transmission

**Fig. 5:**
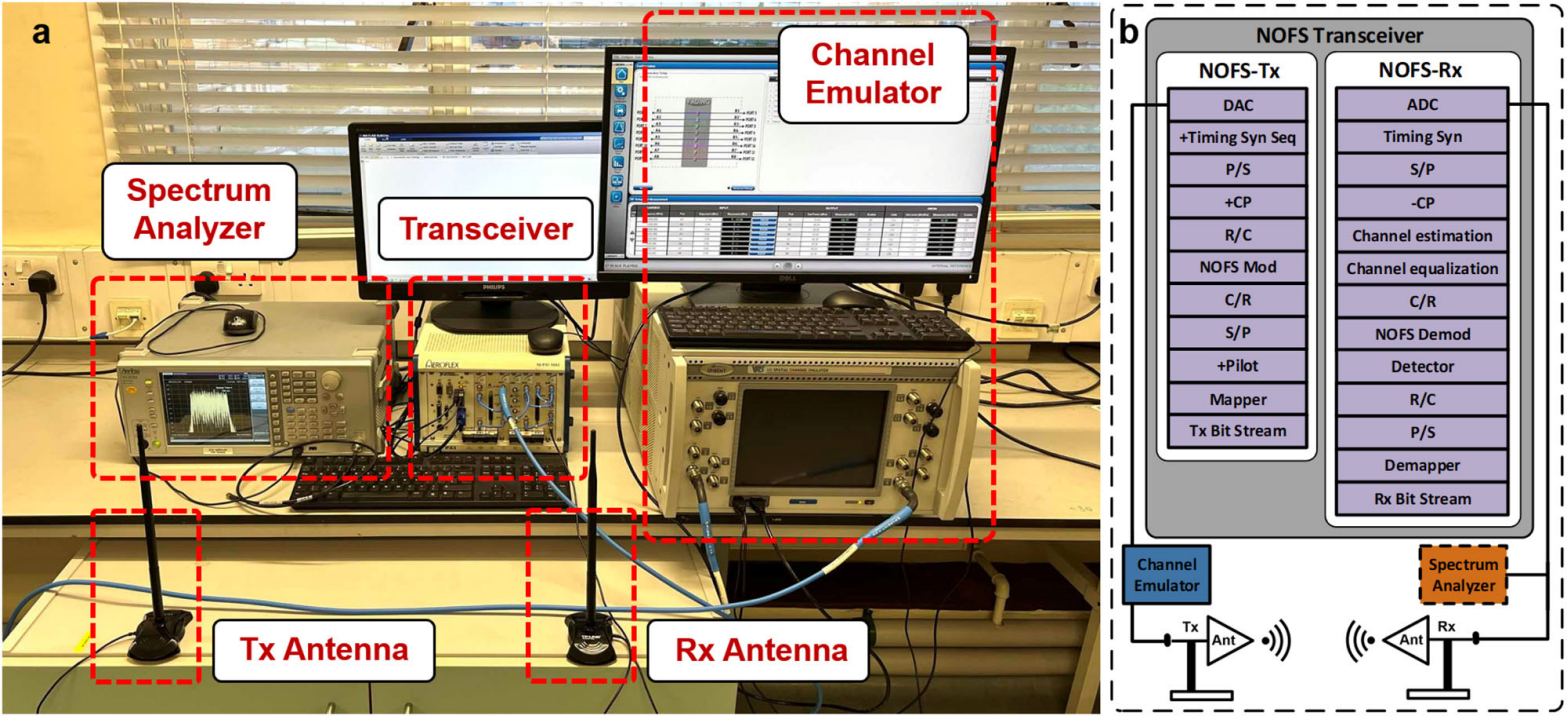
Experiment setup of NOFS over-the-air wireless signal communications. **a** The hardware platform, in which non-orthogonal frequency shaping (NOFS) signals are trained, generated and received. Signals are transmitted over the air via two omni-directional antennas. Signals are distorted via the channel emulator and signal spectral features are measured in the spectrum analyzer. **b** Block diagram of the signal processing within the transceiver. DAC Digital-to-analogue converter, ADC Analogue-to-digital converter, S/P Serial to parallel, P/S Parallel to serial, R/C Real to complex, C/R Complex to real, CP Cyclic prefix.

To validate the designed NOFS waveform in a real world approaching environment, we set up an over-the-air wireless transmission system demonstrated in Fig. 5a. The setup includes a transceiver PCI extensions for instrumentation (PXI) chassis^51,52^, a Spirent VR5 channel emulator^53^, a spectrum analyzer and two omni-directional 8 dBi antennas. The core module is the NOFS transceiver where a transmitter and a receiver are integrated to manage digital signal processing as shown in Fig. 5b. All the signal processing steps are similar to conventional OFDM except for the neural network based NOFS modulation and demodulation. In addition, a particular signal detector is also needed to mitigate ICI for NOFS signals. The details of the NOFS prototyping system configurations are included in Methods.

**Fig. 6:**
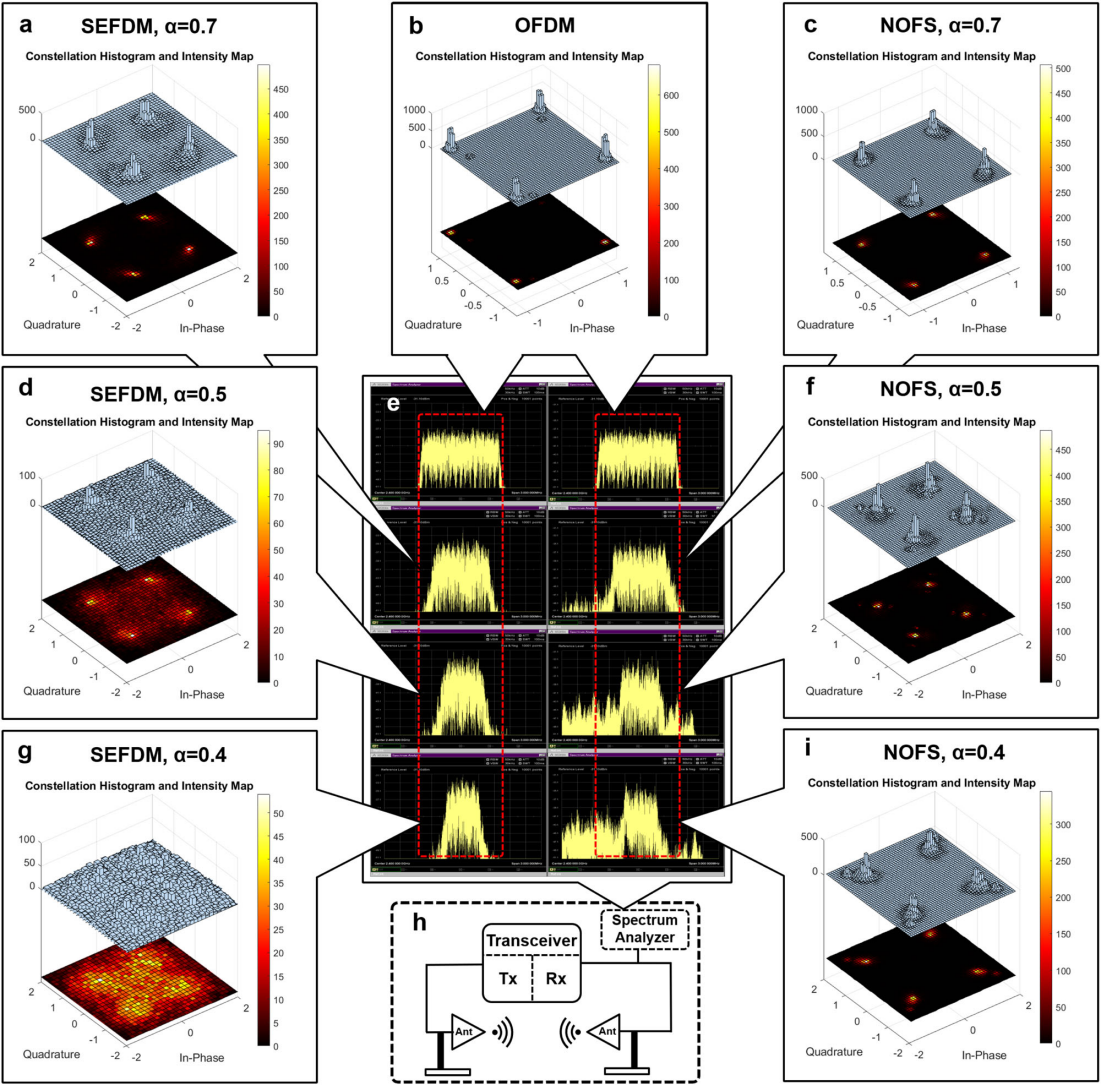
Validation of NOFS signal waveform in receiver side decoded signal constellation performance via over-the-air transmission. This experiment employs quadrature phase shift keying (QPSK) modulation schemes where four constellation points are distributed in the format of histogram and intensity map to indicate information as 1+1i, 1-1i, -1+1i, -1-1i. **a** Constellation of spectrally efficient frequency division multiplexing (SEFDM) at *α* = 0.7. **b** Constellation of orthogonal frequency division multiplexing (OFDM). **c** Constellation of non-orthogonal frequency shaping (NOFS) at *α* = 0.7. **d** Constellation of SEFDM at *α* = 0.5. **e** Spectral comparison for waveforms after over-the-air signal transmission. **f** Constellation of NOFS at *α* = 0.5. **g** Constellation of SEFDM at *α* = 0.4. **h** Block diagram of the experiment setup. **i** Constellation of NOFS at *α* = 0.4.

Over-the-air performance for the NOFS signal transmission is studied at both transmitter and receiver sides, examining spectral and constellation patterns as depicted in Fig. 6. To show the quality of recovered signals, Fig. 6 investigates constellation distributions in terms of histogram and its intensity mapping. A constellation pattern is a representation of a signal in the information format of amplitude and phase. This work tests the basic QPSK constellation pattern where four complex-value constellation points (i.e. 1+1i, 1-1i, -1+1i, -1-1i) are mapped as the information patterns. For SEFDM waveforms, the non-orthogonally packed sub-carriers pose interference to signals and degrade signal performance. In Fig. 6a, constellation points at *α* = 0.7 are still distinguishable as the interference is not significant. As a benchmark, OFDM constellation performance is illustrated in Fig. 6b. The upper layer shows a three-dimensional (3D) histogram with elements centered around constellation points. The lower layer shows a two-dimensional (2D) intensity map projected from the 3D histogram, highlighting the clear separation of information points due to sub-carrier orthogonality, resulting in minimal interference. Our proposed NOFS waveform maintains recoverable information quality, as displayed in Fig. 6c, with distinguishable constellation points similar to the OFDM performance in Fig. 6b. With the decrease of *α* in Fig. 6d, SEFDM constellation patterns at *α* = 0.5 become indistinguishable and symbols are chaotically distributed due to the increasing interference. For non-orthogonal waveforms, with the reduction of *α*, signal spectral bandwidth is compressed accordingly. The SEFDM bandwidth saving is presented in the first column in Fig. 6e, while the second column illustrates the spectral characteristics of NOFS across various bandwidth compression ratios. Our proposed NOFS signal shows robust constellation quality in Fig. 6f even at *α* = 0.5. With further bandwidth decrease to *α* = 0.4 in Fig. 6g, SEFDM constellations are not recoverable. Figure 6h presents a back-to-back transmission evaluation, where the over-the-air communication link is designed with two antennas spacing at 60 cm distance. The spectral analyzer is placed at the Rx side to monitor captured signal spectral characteristics after over-the-air transmission. It should be noted that the quality of recovered constellation patterns from NOFS waveforms at *α* = 0.7,0.5,0.4 in Fig. 6c, f, i are all similar to that of OFDM.

**Fig. 7:**
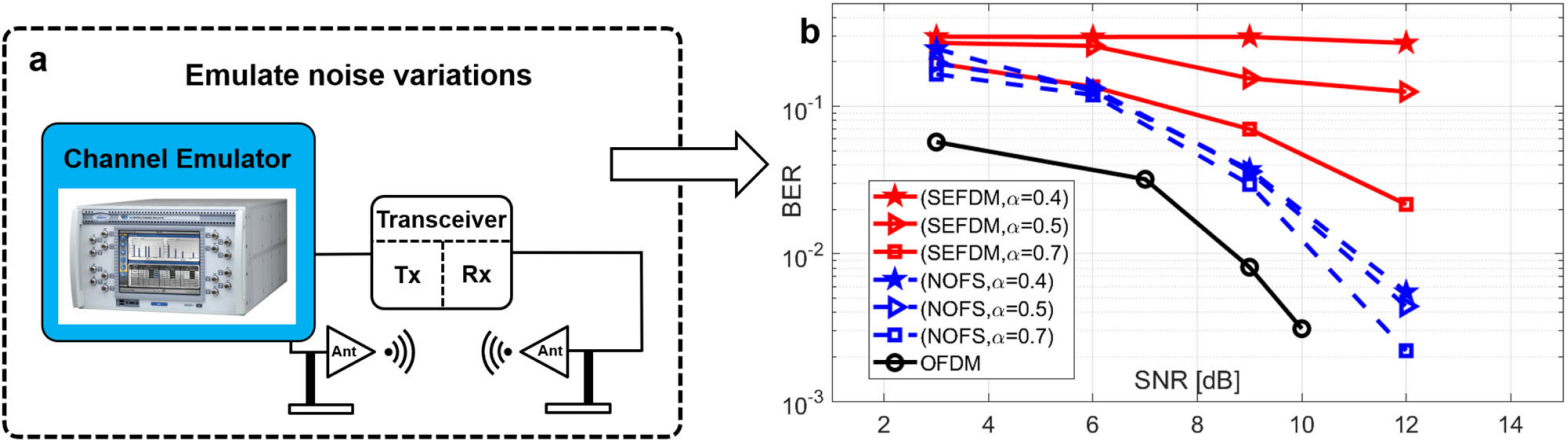
Validation of NOFS signal waveform in BER performance via over-the-air transmission. **a** Block diagram of the experiment setup with channel emulation. **b** Bit error rate (BER) performance with noise variations. OFDM Orthogonal frequency division multiplexing, SEFDM Spectrally efficient frequency division multiplexing, NOFS Non-orthogonal frequency shaping.

To test the new signal format under noise variations, signals are contaminated by additive white Gaussian noise (AWGN) using the VR5 channel emulator in Fig. 7a with BER performance shown in Fig. 7b. The BER performance reveals that NOFS can achieve similar performance with OFDM at different noise power using only 40% of spectral resources while the traditional SEFDM shows error floor. Therefore, it is inferred that achieving the similar performance, this newly proposed NOFS waveform can save 60% of spectral resources (improve 150% spectral efficiency) relative to OFDM.

**Fig. 8:**
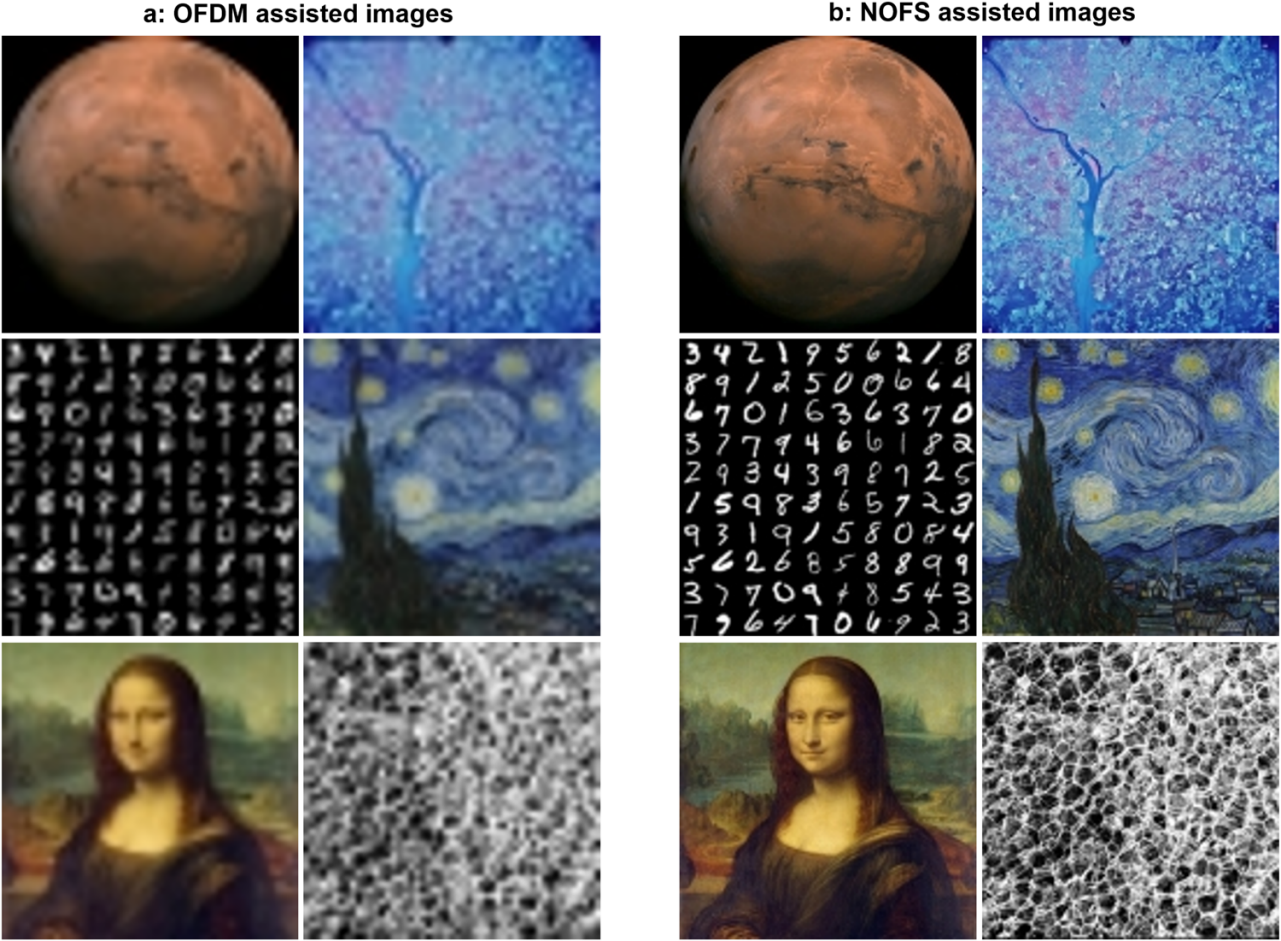
Real-world applications of NOFS signal waveform and the enhanced image resolution advantages. **a** Orthogonal frequency division multiplexing (OFDM) assisted images. **b** Non-orthogonal frequency shaping (NOFS) assisted images. Application scenarios could be in deep space communications, geographical sensing, digit recognition, entertainment and media, face recognition, and medical imaging.

To demonstrate the advantages of our proposed NOFS waveform in practical applications, we successfully transmit and recover images over the air in Fig. 8. We select six sets of images and each set will be evaluated by the OFDM signal in Fig. 8a and our proposed NOFS signal in Fig. 8b. Since NOFS has been verified to have similar performance with OFDM but occupying only 40% of spectral resources, the way to make use of NOFS is to utilize the reserved 60% of spectral resources to deliver extra information. Therefore, given the same spectral resource, NOFS signals can send more pixels indicating higher image resolution. Therefore, the original images supported by OFDM signals have dimensions of 51 × 51 pixels while the NOFS signals can support images with dimensions of 128 × 128 pixels given the same spectral resources. It is clear from Fig. 8 that the NOFS assisted images have much higher resolution than that of OFDM assisted, which can enhance a variety of emerging applications such as deep space communications, geographical sensing, digit recognition, entertainment and media, face recognition, and medical imaging.

## Conclusions

We have reported a non-Nyquist waveform, termed non-orthogonal frequency shaping, designed by an integrated waveform and intelligence framework, that enables more than 25% improvement in spectral efficiency. We also show that the spectral efficiency can be further improved by 100% using a two-dimension modulation format in NOFS, which potentially achieves beyond another waveform limit termed Fast-OFDM where it reported doubling spectral efficiency only when one-dimension modulation pattern is applied. Furthermore, we demonstrate a new record of spectral efficiency limit using NOFS with 150% spectral efficiency improvement, which has never been reached by other waveforms.

Our approach is fit for future diverse 6G services since fundamental physical layer air waveform design enables a great variety of applications. Firstly, this new waveform can find applications in long distance resource-constrained IoT communications where signal spectral resources are limited. In addition, NOFS could also benefit high frequency mmWave and THz communications where point-to-point links using directional narrow beams are utilized. In this case, the spectral resource saving in NOFS can lower the hardware requirement especially the bandwidth requirement for mmWave and THz components, which is beneficial to 6G sustainability where low-cost, power efficient and green motivated techniques are key factors to cut carbon emission. We practically validated the proposed waveform in hardware communication testbed with over-the-air image transmission. Results show higher resolution image transmissions are achieved by NOFS due to 150% more image pixels are able to be sent relative to the OFDM transmission capacity.

## Methods

### irSinc design methodology

Considering breakthroughs in new signal waveform design, we didn’t follow the traditional information-theoretic approach to design and optimize signals. Instead, we employ advanced machine learning techniques to fundamentally create inexistent irSinc to cut signal internal interference and lead to the new waveform design breakthrough. Therefore, multiple irSinc shapes have to be jointly optimized to reach globally optimal performance. The irSinc is irregular and changeable such that a universal and mathematically achievable formula is not derived in this work to define the signal. In this work, only the neural network architecture is utilized to define the signal model. In this case, we cannot provide an information-theoretic formula for our proposed signal and its information-theoretic capacity is not available either. Fortunately, the shallow neural network for the NOFS signal generation is explainable and provides a deterministic communication system architecture for signal generation. Using our practical engineering design approaches, which are not information theoretic based, we successfully validate the NOFS signals using over-the-air transmission experiment, and have shown the performance similarity between experiment results and simulation results.

### Signal detection models

To simplify signal processing expression, the matrix formats for signal generation at Tx and reception at Rx are presented below

9 $$Transceiver=\left\{\begin{array}{l}Tx:\,\,{{{{{{{\bf{x}}}}}}}}={{{{{{{\bf{F}}}}}}}}{{{{{{{\bf{s}}}}}}}}\\ Rx:\,\,{{{{{{{\bf{r}}}}}}}}={{{{{{{\bf{{F}}}}}}}^{* }}}{{{{{{{\bf{x}}}}}}}}+{{{{{{{\bf{{F}}}}}}}^{* }}}{{{{{{{\bf{z}}}}}}}}={{{{{{{\bf{C}}}}}}}}{{{{{{{\bf{s}}}}}}}}+{{{{{{{{\bf{z}}}}}}}}}_{{{{{{{{\bf{{F}}}}}}}^{* }}}}\end{array}\right.$$

where **x** is the *N*-dimensional vector of transmitted samples, **s** is the *N*-dimensional vector of raw symbols, **F** is the sub-carrier matrix with element denoted as $\exp \left(\frac{j2\pi nk\alpha }{N}\right)$. At the receiver side, **r** is the *N*-dimensional vector of demodulated symbols, **F** ^*^ is the demodulation sub-carrier matrix, **z** indicates AWGN and **C** = **F** ^*^ **F** is the correlation matrix between modulation sub-carriers and demodulation sub-carriers. For orthogonal signals, **C** only has diagonal elements indicating no interference from adjacent sub-carriers. However, for non-orthogonal signals, **C** has both diagonal and non-diagonal elements indicating ICI from other sub-carriers.

To achieve OFDM approaching performance, the optimal maximum likelihood (ML) detection has to be used to remove the non-diagonal interference from adjacent sub-carriers. Its operation is expressed as

10 $${\bf{s}}_{ML}=\arg \mathop{\min}\limits_{{\bf{s}}\in {O}^{N}}{\left\Vert {\bf{r}}-{\bf{C}}{\bf{s}}\right\Vert}^{2},$$

where *O* is the modulation cardinality and *O* ^*N*^ indicates the entire candidate solutions. The objective of (10) is to search all possible solutions in **s** ∈ *O* ^*N*^ and find out the best solution that minimizes the term ${\left\Vert {{{{{{{\bf{r}}}}}}}}-{{{{{{{\bf{C}}}}}}}}{{{{{{{\bf{s}}}}}}}}\right\Vert }^{2}$. It is apparent that the complexity is exponentially increased with the increase of modulation cardinality *O* and the number of *N*.

The sub-optimal iterative detection (ID) method^43^ provides a trade-off between performance and processing complexity. The principle of ID is based on iterative interference cancellation and it is mathematically expressed as

11 $${{{{{{{{\bf{s}}}}}}}}}_{\zeta }={{{{{{{\bf{r}}}}}}}}-({{{{{{{\bf{C}}}}}}}}-{{{{{{{\bf{e}}}}}}}}){{{{{{{{\bf{s}}}}}}}}}_{\zeta -1},$$

where **s**_*ζ*_ is the symbol vector after *ζ* iterations, **s**_*ζ*−1_ indicates the detection results after *ζ* − 1 iterations and **e** is an *N* × *N* identity matrix.

### NOFS neural modulator training

As illustrated in Fig. 2, the entire training framework includes two stages. It is noted that the classical Mazo theory is on the basis of uncoded non-orthogonal signals, and therefore we don’t consider channel coding in the training framework. The aim of this work is to investigate the possibility to achieve higher spectral efficiency than the classical Mazo theory by exploring fundamentally new signal features rather than relying on the error correction capabilities from channel coding.

At stage-I, a fully connected neural network, based on the principle in (5), is trained to approximate signals using the training data. To ensure a low-complexity architecture, the neural network at Stage-I is designed without any hidden layers and only input and output layers are stacked. For the purpose of simplification, this work configures signals with *N* = 16 sub-carriers. In neural network training, real values are commonly used instead of complex values. Therefore, the neural network input/output dimension is 2*N* = 32, which consists of 16 real components and 16 imaginary components. There are 16,000 QPSK symbols being selected to train the neural network at Stage-I. The learning rate is 0.01 and the training loss is calculated based on mean squared error (MSE). To reserve signal features, we use linear activation functions *σ*( ⋅ ). In addition, the neural network set the bias Ψ equals zero in order to have the similar signal processing complexity with (2). The elements in the matrix **W** indicate the weights for each neural connection. Random values are initially given to the elements in **W** and the optimal values will be obtained after a number of training iterations via back propagation using the stochastic gradient descent (SGD) optimization algorithm. To maximize the error performance or in other words to maximize the signal to interference power ratio, the irSinc has to be optimized for each specified signal configuration. When the values of signal compression factors are changed, the signal interference characteristic will be changed accordingly and therefore we need to update **W** and re-optimize all the irSinc to maximize the error performance.

At Stage-II, the fully connected neural network weight matrix **W**, obtained from the Stage-I training, is directly used. A sparse neural network is obtained after discarding neural connections associated with small weights. A pruning factor *γ*, is introduced to determine the number of discarded neural connections with the value defined as *γ* = 1, 2, . . . , *N* ^2^. For example, the initial value is set to *γ* = 1 indicating one discarded neural connection while *γ* > 1 indicating more neural connections are discarded. After each neural connection pruning, the sparse neural network is re-trained together with the original training data to simultaneously keep neural network pruning advantages and original signal features. It is noted that the re-training at Stage-II is not compulsory and the sparse neural network can work well even without the re-training operation. For each value of *γ*, a matrix **W**_**γ**_ is obtained to modulate the symbol vector **s** following the process in (6). To ensure convincing performance analysis, 800,000 QPSK symbols are used for the BER testing at Stage-II. Since the output from the sparse neural network is in a real-value format, the signal has to be converted to its complex-value format in the R/C block before transmitting through the channel. To have a robust neural modulator for NOFS, we test the NOFS modulated waveform in AWGN under a wide range of Eb/N0 covering [1, 2, . . . , *β*] dBs. After the channel distortion, the complex-value signals are converted back to its real-value format in the C/R block. For each Eb/N0, the AWGN distorted NOFS signal is demodulated by ${{{{{{{\bf{{W}}}}}}}_{\gamma }^{* }}}$. The demodulated signal is then fed to the signal detector for ICI cancellation. It is noted that in addition to the iterative detection method, other detection approaches are also applicable. After the signal detection, the Stage-II framework computes BER for the detected symbols at each Eb/N0. BER is the important optimization metric in this work to search for the optimal value of *γ*. At the final step, MSE is computed for a vector of BER ranging from Eb/N0 = 1 dB to Eb/N0 = *β* dB, under a specific value of *γ*. The process is repeated by increasing the value of *γ* for each tuning of **W**_**γ**_. The optimal solution is obtained when the smallest MSE value is reached.

### NOFS prototyping hardware configurations

The prototyping testbed is designed and operated indoor as shown in Fig. 5a. The designed signal is transmitted over the air at 2.4 GHz, which is a license free carrier frequency. The indoor space is limited and two 8 dBi omni-directional antennas are placed at 60 cm distance which is a far-field range^54^. The output signal from the NOFS-Tx is −17 dBm. The 11.24 cm signal wavelength effectively causes signal multipath propagation in an indoor space but the effect is ‘weak’ due to the close spacing of the two antennas. Fortunately, the ‘weak’ frequency selective channel ensures fair comparison between OFDM and NOFS. Otherwise, the bandwidth compressed NOFS signal can use the spectral scheduling mechanism to dynamically select high quality frequency band and squeeze in a better channel, where the performance improvement for the scheduling scheme has been experimentally validated in^55^. To compensate for the short distance indoor transmission, the VR5 channel emulator is applied to introduce power attenuation and AWGN. The VR5 is also configured to work at 2.4 GHz and the noise bandwidth is set to 6 MHz and the receiver bandwidth is set to 3.125 MHz. To emulate power loss during practical transmission, VR5 introduces 8 dB power degradation. To have noise variations, VR5 adjusts noise power from −37 dBm to −28 dBm. Therefore, the input power (output power from NOFS-Tx) to the VR5 is −17 dBm, the output power from the VR5 is −25 dBm, and the signal-to-noise ratio (SNR) ranges from 3 dB to 12 dB. In this case, we can plot the constellation patterns and BER at different SNR. A spectrum analyzer is placed at the receiver to monitor received signal spectral characteristics in Fig. 6e where the measurement is operated with back-to-back antenna transmission.

The signal processing for NOFS waveform transmission is illustrated in Fig. 5b. At NOFS-Tx, a random bit stream is generated and mapped to a modulation format such as QPSK in this work. In practice, signal distortions exist and pilots are required for channel estimation, imperfect timing and phase equalization. After the serial to parallel (S/P) conversion, the mapped symbols are ready for NOFS signal generation. The complex to real (C/R) conversion is required because real values have to be used for the subsequent neural network signal generation. After the NOFS modulation following the principle in Fig. 2, the real value samples are real to complex (R/C) converted. To avoid ISI and multipath interference, cyclic prefix (CP) is added at the beginning of each NOFS symbol. It is noted that the benefit of CP is to simplify channel estimation and equalization into one-tap operations subject to the use of fast Fourier transform (FFT) and inverse fast Fourier transform (IFFT) at the transmitter and the receiver respectively. Since NOFS waveform removes FFT and IFFT, the use of CP might not be very beneficial but occupying extra power. An alternative option for NOFS is to use zero padding (ZP) where an empty protection gap is reserved leading to higher power efficiency. To be compatible with conventional waveform generation methodology and to have fair comparisons, we still use CP in this work. After the parallel-to-serial (P/S) conversion, a timing synchronization sequence is added at the beginning of the data sequence. So far, the digital processing for the transmitter side signal is complete. After the digital-to-analogue converter (DAC) module, analog signals are obtained and distorted by the channel emulator. For over-the-air transmission experiment, two omni-directional 8 dBi antennas are placed in line of sight. Due to the limited indoor space, in order to test and compare signal waveforms at different noise levels, the channel emulator VR5 is used to emulate noise and distort signals. In this experiment, due to the signal characteristics and limitations of the PXI chassis and the VR5 channel emulator, we configure the OFDM signal bandwidth at 3 MHz and the bandwidths for SEFDM and NOFS are narrower due to spectral bandwidth compression.

At NOFS-Rx, analog signals are captured via the Rx antenna and sent to analogue-to-digital converter (ADC) for conversion. The raw digital signals are then timing synchronized to locate the accurate starting position of the data sequence. The serial data stream is converted to a parallel format for the purpose of symbol-by-symbol signal processing. After removing CP, a time-domain channel estimation algorithm is operated using the NOFS pilot symbols to obtain the wireless channel information, which is later used to equalize distorted NOFS data symbols. Complex symbols are converted to real symbols for the purpose of NOFS demodulation because only real values are acceptable by the NOFS neural network. Following the demodulation, real symbols are interference mitigated within the signal detection block. After R/C and P/S operations, the recovered complex symbols are demapped to bit sequences for performance comparisons.

### Advanced signal setup

**Fig. 9:**
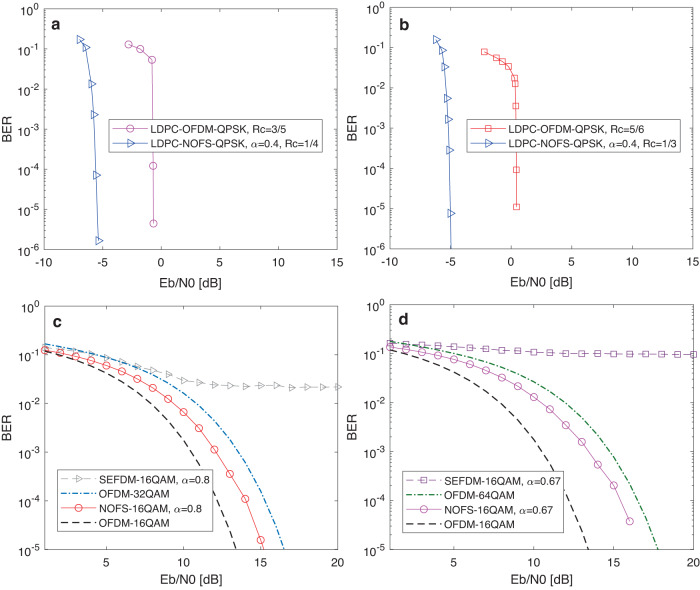
Performance investigations for coding and high order modulation based NOFS signals. **a** LDPC coding, effective spectral efficiency 1.25 bit/s/Hz. **b** LDPC coding, effective spectral efficiency 1.67 bit/s/Hz. **c** 16QAM-NOFS, *α* = 0.8, spectral efficiency 5 bit/s/Hz. **d** 16QAM-NOFS, *α* = 0.67, spectral efficiency 6 bit/s/Hz. OFDM Orthogonal frequency division multiplexing, SEFDM Spectrally efficient frequency division multiplexing, NOFS Non-orthogonal frequency shaping, QPSK Quadrature phase shift keying, QAM Quadrature amplitude modulation, LDPC Low density parity check.

Channel coding plays important roles in error corrections in communication systems and therefore more results for low density parity check (LDPC) coded NOFS signals are evaluated here. To have convincing results, we use the LDPC coding framework from Matlab toolbox where the LDPC coding follows the digital video broadcast (DVB) standard. The coding bit sequence has 64,800 bits and the number of information bits and parity bits are determined by a coding rate. In this work, we comprehensively test the coding rates of 1/4, 1/3, 3/5, 5/6. To show the effectiveness of our proposed NOFS waveform, we include the performance of OFDM as the benchmark. The main advantage of NOFS is its increased spectral efficiency since its signal transmission spectral resource is saved while data rate is maintained. When channel coding is considered, the enhanced spectral efficiency from NOFS allows more powerful coding schemes. Signals in Fig. 9a, b have the same data rate but NOFS achieves higher spectral efficiency due to the compression of spectral bandwidth. As shown in Fig. 9a, we consider a QPSK modulated LDPC-OFDM system with coding rate of 3/5, which indicates the effective spectral efficiency of 2 × 3/5 = 1.2 bit/s/Hz. This work considers coding rates from the DVB standard. Therefore, to achieve the similar effective spectral efficiency in NOFS (*α* = 0.4), the coding rate is configured to be 1/4, which has the effective spectral efficiency of 2 × 1/4 × (1/0.4) = 1.25 bit/s/Hz. It is obvious that LDPC-NOFS can achieve nearly 5 dB performance gain with additional 0.05 bit/s/Hz spectral efficiency gain over LDPC-OFDM even using a matched filtering detector. Therefore, it is inferred from Fig. 9a that the LDPC-NOFS outperforms the traditional LDPC-OFDM in both BER performance and spectral efficiency. Another example is shown in Fig. 9b where the NOFS at coding rate of 1/3 has the same effective spectral efficiency (i.e. 2 × 1/3 × (1/0.4) = 1.67 bit/s/Hz) with the OFDM at coding rate of 5/6 (i.e., 2 × 5/6 = 1.67 bit/s/Hz). The advantage is apparent that the LDPC-NOFS can achieve 6 dB performance gain over the LDPC-OFDM.

High order modulation patterns are commonly used in wireless communications to enhance spectral efficiency. Here we test 16QAM modulated NOFS signals with different compression factors, and compared with 16QAM, 32QAM and 64QAM modulated OFDM signals. In addition, 16QAM modulated SEFDM is included as a benchmark. The neural network signal generator for 16QAM has to be re-trained in this work due to the change of bandwidth compression factors and symbol decision solutions. It is noted that NOFS in Fig. 9c, d has lower data rate compared to OFDM due to the use of lower order modulation format. However, NOFS achieves the same spectral efficiency with OFDM because of the compression of spectral bandwidth. In Fig. 9c, it is obvious that 16QAM modulated SEFDM fails due to the interference from high modulation density and signal internal ICI. The 16QAM modulated NOFS signal with *α* = 0.8 is worse than 16QAM modulated OFDM but it outperforms the spectral efficiency equivalent 32QAM modulated OFDM signal even using a zero forcing detector. Further reducing the compression factor to *α* = 0.67, similar trends are observed in Fig. 9d where 16QAM modulated SEFDM signal fails and the 16QAM modulated NOFS signal with *α* = 0.67 outperforms spectral efficiency equivalent 64QAM modulated OFDM signal. Therefore, low order modulation based NOFS signal can replace high order modulation based OFDM signals with even better BER performance.

## Data Availability

The data that support the experiment of this work are available from the corresponding author upon request.

## Code availability

The codes that generate the results are available from the corresponding author upon request.
